# Polymorphisms in Phase I and Phase II genes and breast cancer risk and relations to persistent organic pollutant exposure: a case–control study in Inuit women

**DOI:** 10.1186/1476-069X-13-19

**Published:** 2014-03-16

**Authors:** Mandana Ghisari, Hans Eiberg, Manhai Long, Eva C Bonefeld-Jørgensen

**Affiliations:** 1 Centre for Arctic Health & Unit of Cellular and Molecular Toxicology, Department of Public Health, Aarhus University, Bartholins Álle 2, Build 1260, 8000 Aarhus C, Denmark; 2 Department of Cellular and Molecular Medicine, Panum Institute, University of Copenhagen, Copenhagen, Denmark

**Keywords:** Breast cancer, Inuit, Genetic polymorphism, CYP1A1, CYP1B1, COMT, CYP19

## Abstract

**Background:**

We have previously reported that chemicals belonging to the persistent organic pollutants (POPs) such as perfluorinated compounds (PFAS) and polychlorinated biphenyls (PCBs) are risk factors in Breast Cancer (BC) development in Greenlandic Inuit women. The present case–control study aimed to investigate the main effect of polymorphisms in genes involved in xenobiotic metabolism and estrogen biosynthesis, CYP1A1, CYP1B1, COMT and CYP17, CYP19 and the BRCA1 founder mutation in relation to BC risk and to explore possible interactions between the gene polymorphisms and serum POP levels on BC risk in Greenlandic Inuit women.

**Methods:**

The study population consisted of 31 BC cases and 115 matched controls, with information on serum levels of POPs. Genotyping was conducted for CYP1A1 (Ile462Val; rs1048943), CYP1B1 (Leu432Val; rs1056836), COMT (Val158Met; rs4680), CYP17A1 (A1> A2; rs743572); CYP19A1 (C> T; rs10046) and CYP19A1 ((TTTA)n repeats) polymorphisms and BRCA1 founder mutation using TaqMan allelic discrimination method and polymerase chain reaction based restriction fragment length polymorphism. The χ^2^ –test was used to compare categorical variables between cases and controls and the odds ratios were estimated by unconditional logistic regression models.

**Results:**

We found an independent association of CYP1A1 (Val) and CYP17 (A1) with BC risk.

Furthermore, an increased BC risk was observed for women with high serum levels of perfluorooctane sulfonate (PFOS) and perfluorooctanoic acid (PFOA) and carriers of at least: one CYP1A1 variant Val allele; one variant COMT Met allele; or the common CYP17 A1 allele. No combined effects were seen between PFAS exposure and CYP1B1 and CYP19 polymorphisms. The risk of BC was not found significantly associated with exposure to PCBs and OCPs, regardless of genotype for all investigated SNPs. The frequency of the Greenlandic founder mutation in BRCA1 was as expected higher in cases than in controls.

**Conclusions:**

The BRCA1 founder mutation and polymorphisms in CYP1A1 (Val) and CYP17 (A1) can increase the BC risk among Inuit women and the risk increases with higher serum levels of PFOS and PFOA. Serum PFAS levels were a consistent risk factor of BC, but inter-individual polymorphic differences might cause variations in sensitivity to the PFAS/POP exposure.

## Background

Breast cancer (BC) is the most common cancer for women in the western world and the incidence has been increasing since 1940. Known established BC risk factors include genetic inheritance e.g. mutations in the BRCA1 and BRCA2 genes, lifelong exposure to oestrogens (early menarche and late menopause increases the risk), obesity after menopause, alcohol, smoking and high intake of fat (reviewed in [1]). Some factors seem to reduce the risk such as low age at first birth, large number of full term pregnancies and long duration of breastfeeding. However, the known risk factors only explain less than a third of all cases and the risk of BC is thought to be modified by lifestyle and environmental exposures. Previous studies have shown that Inuit had lower incidence of various diseases (e.g. cancer) compared to the Danish population [2]. The incidence of BC has traditionally been low among the Inuit, but since 1970’s a considerable increase has been observed in Greenland and Canada [3,4] to a level approximately 50% of the incidence in e.g. Denmark. This may suggest that changes in lifestyle and environmental factors might be affecting the BC risk possibly in combination with the genetic factors [1].

**Figure 1:**
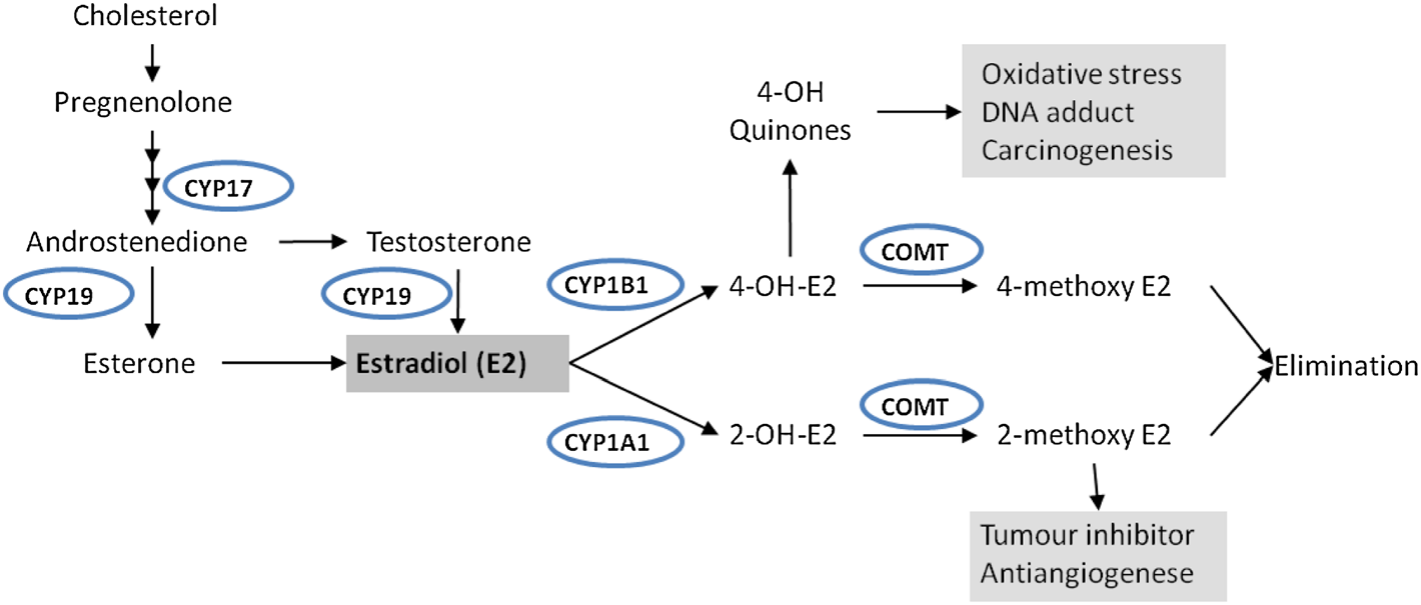
**Simplified schematic presentation of enzymes involved in estrogen biosynthesis and metabolism.** CYP1A1 and CYP1B1 are also involved in metabolism of environmental chemicals. 17β-Estradiol can be metabolised into the hydroxylated catecholestrogens, 2-OHE_2_ and 4-OHE_2_ by CYP1A1 and CYP1B1, respectively. These catecholestrogens can be cleared via the methylation pathway catalysed by COMT. Alternatively the 4-OHE_2_ can be oxidised into the semiquinone, which can undergo non-enzymatic conversion to its quinone generating superoxide radical. E_2_: 17β-estradiol; 2-OHE_2_: 2-hydroxyestradiol; 4-OHE_2_: 4-hydroxyestradiol; COMT: catechol-*O*-methyl transferase.

Polymorphisms including single nucleotide polymorphisms (SNPs) in genes involved in xenobiotic metabolism and in genes involved in oestrogen biosynthesis and metabolism might affect circulating estrogen levels and modulate the individual susceptibility to environmental carcinogens in relation to developing BC [5]. Such candidate genes that have been focused on in the literature and are included in our study are (Figure 1): cytochrome P450 (CYP) 1A1, CYP1B1, Catechol-O-methyltransferase (COMT), 17α-hydroxylase and 17, 20-lyase (CYP17) and aromatase (CYP19).

The CYP1A1 and CYP1B1 are Phase I xenobiotic metabolising enzymes, playing important roles in the detoxification of xenobiotics and metabolism of endogenous steroid hormones (reviewed by [6]). CYP1A1 and CYP1B1 catalyse the conversion of oestradiol to the 2-OH and 4-OH catechol oestrogen metabolites (2-OH-E2 and 4-OH-E2), respectively (Figure 1). The 2-OH-E2 lacks significant oestrogenic activity whereas the 4-OH-E2 is highly estrogenic and carcinogenic in animal models [7] and shows increased expression in neoplastic mammary tissue [8].

Additionally, CYP1A1 and CYP1B1 metabolically activate numerous procarcinogens including environmental contaminants such as polycyclic aromatic hydrocarbons (PAHs), polyhalogenated aromatic hydrocarbons (PHAHs) (e.g. dioxin-like compounds and polychlorinated biphenyls (PCBs) and aryl amines to reactive epoxide intermediates that might increase the risk of oxidative stress and cancer [6,9]. The genes are inducible by some environmental chemicals via the aryl hydrocarbon receptor (AhR) [10,11]. Several SNPs have been identified in CYP1A1, some of which lead to a higher inducible aryl hydrocarbon hydrolase (AHH) enzyme activity [9]. One CYP1A1 SNP includes the A to G transition at position 4889 in exon 7 resulting in a change from an isoleucine to valine amino acid (Ile → Val) at codon 462 [12]. This variant is reported to be significantly associated with CYP1A1 inducibility and higher AHH enzyme activity [9,13] that might cause higher rates of carcinogen activation. For CYP1B1 gene an important SNP (C → G) at codon 432 in exon 3 leads to an amino acid substitution of leucine to valine (Leu → Val), which increases the 4-OH activity of CYP1B1 by 3 fold [14,15]. Due to their role in procarcinogen activation and oxidative metabolism of oestrogens, there have been several investigations on the association of CYP1A1 and CYP1B1 polymorphisms and a variety of oestrogen related diseases e.g. BC and inconclusive results have been reported [16,17].

The COMT, a phase II enzyme, methylates the catechol oestrogens and convert them to their non-genotoxic methoxy derivatives. In the COMT gene, a single G to A base pair change results in an amino acid change from valine to methionine (Val → Met) at codon 108 of the soluble form of COMT and codon 158 of the membrane-bound form of COMT. This amino acid change has been associated with 3- to 4-fold decrease in enzyme activity in vitro [18,19]. Individuals carrying the Met allele are hypothesised to have a decreased ability to form the anti-tumour 2-methoxyestradiol, causing an increased accumulation of the reactive catechol oestrogen intermediates and thus facilitating the development of oestrogen-induced tumours e.g. in BC. Epidemiological study data on the influence of the variant COMT (Met/Met) with human breast cancer are controversial [20,21].

The CYP17A1 gene (CYP17), codes for the cytochrome P450c17α enzyme involved in the early stages of oestrogen biosynthesis by catalysing the 17α-hydroxylation of pregnenolone and progesterone to precursors of androgen and oestrogen, respectively (Figure 1). A common single base pair substitution (−34 T → C) in the 5′ untranslated region (5′-UTR) of CYP17 creates an additional SP1 promoter site which originally was thought to up-regulate CYP17 transcription [22]. However, a later study did not support this hypothesis [23] and the functional impact of the T/C change is presently to our knowledge not known. The common T allele is referred as A1 and the variant C allele as A2. The rare allele A2, compared to A1, is postulated to correlate with higher serum levels of various sex steroids in some studies (e.g., testosterone, progesterone, oestrone, and oestradiol) [22,24,25]. To date, several studies have reported the association between CYP17 polymorphism (−34 T → C) and BC although with conflicting results (ref. in [26]).

The CYP19A1 (CYP19) gene encodes aromatase, the enzyme that catalyses the final step of oestrogen biosynthesis by converting androgens (testosterone and androstenedione) to oestrogenic steroids (oestradiol and oestrone) (Figure 1) [26]. Polymorphisms at this locus may affect gene activity and thereby affect hormone levels. Two of the polymorphisms, the tetra-nucleotide repeat ([TTTA]n) polymorphism which varies from 7 to 13 repeats in intron 4 and a C → T single nucleotide polymorphism (SNP) (rs10046) in the 3′-UTR of the CYP19 gene, have been frequently studied for BC associations with inconsistent results. The variant allele of rs10046 SNP (T) has been associated with higher levels of postmenopausal circulating E2 [27]. The relatively longer alleles of CYP19 (TTTA)_n_ polymorphism such as (TTTA)_10_, (TTTA)_11_ and (TTTA)_12_, have been associated with increased risk for development of BC in some studies [28]. There have been reported some associations of one or more repeats of (TTTA)_8_ or (TTTA)_7,_ with higher circulating oestrogens, but the results are conflicting and inconclusive (reviewed in [29]).

The relationship between SNPs in these genes involved in oestrogen biosynthesis and metabolism and the risk of BC have been investigated in different populations, but to our knowledge no studies have been reported for Inuit who are genetically different from Caucasians. Several studies have reported potential interactions between PCB concentrations and CYP1A1 gene polymorphism associated with increased risk of BC in Caucasians [30-32]. Studies on the gene and gene-environment interaction on cancer risks in Greenlandic Inuit are scarce. Therefore, we hypothesised that the risk of BC is likely to be influenced by polymorphisms in genes related to xenobiotic and oestrogen metabolism by interaction with relevant environmental exposure.

The present investigation is a further analysis of our recent BC case–control study among Greenlandic Inuit women that examined the serum levels of persistent organic pollutants (POPs) including perfluoroalkylated substances (PFASs), polychlorinated biphenyls (PCBs), and organochlorine pesticides (OCPs), in relation to BC risk [33]. An increased BC risk was observed for cases having significantly higher serum level of PFASs and also PCBs in the highest quartile [33].

The present study aimed to investigate polymorphism in the genes CYP1A1, CYP1B1, COMT, CYP17 and CYP19 in relation to BC risk and to explore possible interactions between the polymorphisms and serum POP levels on BC risk in Greenlandic Inuit women.

## Methods

### Study population

This study is based on our recent case–control study of environmental exposure to POPs including PFASs and BC risk in Greenlandic Inuit women; a detailed description of the study population has been reported previously [33]. Briefly, 31 BC cases (equals to 80% of all BC cases during the sampling period 2000–2003) were frequency-matched by age (≤50, 51–55, 56–59,≥60 yrs) and districts of residence with controls (n = 115). All subjects were of Greenland Inuit descent, defined as being born and having more than two grandparents born in Greenland. The sampling period was from 2000–2003 with subjects from Nuuk, Upernavik, Qeqertarsuaq, Narsaq, Tasiilaq, Qaqortoq, Sisimiut, Assiaat, and Nanortalik. Blood samples were taken when the BC was diagnosed. Information was collected through validated Danish standard questionnaires and included questions about demographic and lifestyle parameters and allowed to document the following risk factors for BC: age, breastfeeding, Body Mass Index (BMI), smoking, menopause status. Furthermore, following information were available as described [33]: plasma fatty acids, serum cotinine and serum oestradiol (E2).

The study was approved by Greenlandic Ethic Committee.

### Measurement of plasma legacy POPs and serum PFASs

As described previously [33], for cases the blood samples were taken when the BC was diagnosed, before any treatment. The lipophilic plasma legacy POPs (12 PCB congeners and 8 OCPs), PFASs (seven PFASs including perfluorooctane sulfonate (PFOS) and perfluorooctanoic acid (PFOA)) and lipids were measured as previously given [33]. The analyses of legacy POPs were performed at the Centre de Toxicologie of the Institut National de Santé Publique du Québec (Québec, Canada) (CTQ) and PFASs were measured at the National Environmental Research Institute, Aarhus University, Denmark [33].

The measured PCBs and OCPs concentrations were lipid adjusted and grouped as sum-PCBs and sum-OCPs (μg/kg lipid). Due to difference in chemical and toxicological properties of PCBs, we also conducted the analyses for dioxin-like PCBs as group, but since the results resembled those for sum-PCBs, only the results for sum-PCBs are given. The analysed PFASs were grouped into perfluorosulfonated acids (sum-PFSA) and perfluorocarboxylated acids (sum-PFCA) but the results resembled those of PFOS and PFOA alone, respectively. Therefore, data for sum-PFSA and sum-PFCA are not presented.

### DNA isolation and genotyping

Genomic DNA was extracted from whole blood using the QIAamp DNA mini Kit (Qiagen) according to the manufacturer’s recommendations.

The following SNPs were analysed: CYP1A1 Ile462Val (rs1048943), CYP1B1 Leu432Val (rs1056836), COMT Val158Met (rs4680), CYP17 -34T>C (rs743572), CYP19 C > T (rs10046), CYP19 (TTTA)n repeat and BRCA1 founder mutation (p. Cys39GlY). Table 1 shows the specifications of these polymorphisms. All the genotypes except for BRCA1 and CYP19 (TTTA)n repeat were obtained using TaqMan Drug Metabolism Genotyping Assays, following manufacturer’s instructions (Applied Biosystems, ABI). The TaqMan assay was done in a 12 μL reaction volume containing 1x TaqMan Drug Metabolism Genotyping Assay Mix (containing premixed PCR primers and MGB probes), TaqMan Universal PCR Master mix, and 10–15 ng genomic DNA as described previously [34]. Allele discrimination analyses were performed on the ABI Prism 7000 Sequence Detection System. Positive controls consisting of DNA representing wild-type/wild-type (wt/wt), wt/variant and variant/variant genotypes and negative controls (no DNA) were included in each assay.

**Table 1:** Characteristics of the studied gene polymorphisms

**Gene**	**Position**	**Reference SNP**	**Location/amino acid change**	**Genotyping method**	**Main function**	**Activity**	
						**Low**	**High**
*CYP1A1*	15q24.1	rs1048943	A> C	C_25624888_50^#^	Carcinogen activation	Ile (wt)	Val (var)
			Ile462Val		2-hydroxylation of E2		
*CYP1B1*	2p22.2	rs1056836	C> G Leu432val	C_3099976_30^#^	Carcinogen activation	Leu (wt)	Val^a^ (var)
					4-hydroxylation of E2		
*COMT*	22q11.21	rs4680	G> A	C_25746809_50^#^	Estradiol catabolism	Met (mut)	Val (var)
			Val158Met				
*CYP17A1*	10q24.3	rs743572	5′UTR	C_2852784_30^#^	Steroid 17α-hydroxylase		A2^b^
			−34 T> C (A1> A2)				
*CYP19A1*	15q21.1	rs10046	3′UTR	C_8234731_30^#^	Aromatase activity (androgen to estrogen)		T^c^ (var)
			C> T				
*CYP19A1*	15q21.1	-	(TTTA)n repeats	PCR-based	Aromatase activity	-	-
*BRCA1*	17q21	rs80357164	Cys39Gly	PCR-based	DNA damage repair	-	-

wt: wild-type; var: variant.

^#^TaqMan Drug Metabolism Genotyping Assay.

^a^432Val variant has the higher 17β-estradiol hydroxylation activity, but lower carcinogenic activity [14].

^b^A2 is postulated to correlate with higher serum levels of various sex steroids [22,24,25].

^c^Variant alleles T has been associated with higher levels of postmenopausal circulating E2 [27].

The BRCA1 p.Cys39Gly mutation genotyping was performed using allele-specific PCR amplification with mutation-specific forward primers: BRCA1-39-N-F: 5′-ATCAAGGAACCTGTCTCCACAAAcT-3′ (normal) and BRCA1-39-S-F: 5′-AGGAACCTGTCTCCACAAAcg-3′ (mutation) and reverse primer BRCA1-39-fl-R: 5′-TGACCGGCAGCAAAATTGC TCCTGGGTTATGAAGGACAAA-3′ and a Fam-primer FAM6-TGACCGGCAGCAAAATTGC.

PCR amplifications were carried out in a 15 μL reaction mixture containing 50 ng DNA, 5 μM of each forward primer and 4 μM of the reverse primer, 0.5 unit of Amplicon Taq DNA Polymerase (Bie & Berntsen, Copenhagen, Denmark) and 3 μl 60% sucrose. PCR conditions consisted of an initial melting step for 5 min at 98°C followed by 30 cycles of 98°C for 30 s, 56°C for 45 s and 72°C for 45 s (40 cycles), a final extension step at 72°C for 5 min and a cooling phase to 14°C. The fragments were analysed using ABI 3100: 1 μl PCR product, 0.5 μl 600 size standard and 10 μl formamid. A positive and a negative control were included in each run.

The CYP19A1 genotyping was performed using PCR amplification with the primers 5′- TACAGTGAGCCAAGGTCGTG-3′ (forward) and 5′- AGTGCATCGGTATGCATGAG-3′ (reverse). Fifty ng of DNA in a 15 μl reaction containing 10 μM primers, 0,1 μl Taq DNA polymerase, 1.5 μl Taq polymerase (VWR) was amplified using a touch down PCR program. The PCR condition included a 5 min initial denaturing step at 96°C, 19 cycles amplification (30 s at 96°C, 30 s at 65°C (−0.5°C per cycle) and 30 s at 72°C) and 19 cycles amplification (30 s at 96°C, 30 s at 50°C and 30 s at 72°C), a 72°C extension for 5 min and cooling phase to 14°C.

PCR products were separated on a 2.5% agarose gel containing ethidium bromide.

The number of repeats was not confirmed by sequencing. For statistical analyses, the bands on the gel were categorised as: short (fast) = 7–10 repeats and long (slow) = 11 and more repeats.

The most common repeats reported in the literature are 7 (50%), 8 (10%), 11 (30%), 12 (<5%) [28].

Approximately ten percent of the samples were genotyped in duplicate, and when uncertainties were discovered the genotyping were repeated for these samples (very few samples).

The SNPs in CYP1A1 Ile462Val (rs1048943), CYP1B1 Leu432Val (rs1056836), COMT Val158Met (rs4680), CYP17 -34T>C (rs743572), CYP19 C > T (rs10046), CYP19 (TTTA)n repeat and BRCA1 were successfully genotyped for 98%, 97%, 99%, 98%, 99%, 90% and 95% of the total samples, respectively. Thus, the numbers reported for the different polymorphisms may vary from the total.

### Statistical analysis

Hardy-Weinberg equilibrium (HWE) was tested for the different genotype frequencies using the χ^2^ –test. The χ^2^ –test or Fisher’s exact tests were used to compare categorical variables between cases and controls (allele and genotype frequencies, smoking status, breastfeeding etc.). Fisher’s exact test was also used to compare the genotype differences in the dominant model: common, wild-type (wt) homozygous (CYP1A1: Ile/Ile; CYP1B1: Leu/Leu; COMT: Val/Val; CYP17: A1A1; CYP19 C > T: CC; CYP19 TTTA: (TTTA)_<10_) vs. those with at least one variant (var) allele (CYP1A1: Ile/Val + Val/Val; CYP1B1: Leu/Val + Val/Val; COMT: Val/Met + Met/Met; CYP17: A1A2 + A2A2; CYP19 C > T: CT + TT; CYP19 TTTA: (TTTA)_≥10_).

Unconditional logistic regression analysis was used to estimate odds ratios (ORs) and 95% confidence intervals (CI) for the association between SNPs and BC, and the interaction between SNPs, POP variables and BC risk. For SNP analyses, genotypes were coded as counts of the risk allele (wt/wt = 0; wt/var = 1 and var/var = 2) and the homozygous common (wt/wt) genotype was used as reference group. The genotyping data were also categorised into two groups (dominant model): homozygous common (wt/wt; referent) and heterozygous + homozygous variant alleles (wt/var + var7var). The heterozygous and homozygous variant alleles were combined in order to ensure sufficient sample size for comparisons.

For the association between SNPs and BC, the model was adjusted for age and serum cotinine as continuous variables, since smoking can affect the gene expression of some of the CYP450 genes [9]. We also conducted stratified analysis to see if the association with SNPs varied by menopausal status. Menopausal status was unknown for 36 of the samples (11 cases and 26 controls): among these, 31 women were between 18–42 years old and 5 women between 66–80 years old [33]. In this study, women who had missing information on menopausal status were designated premenopausal if they were between 18–42 years old, and considered postmenopausal if they were between 66–80 years old.

The POP variables were natural logarithmic transformed to improve the normality and homogeneity of variance and thus the analyses were performed on the ln-transformed POP data.

For the interaction between SNPs and POP exposure the model was adjusted for age and cotinine and not with additional factors (e.g. BMI, total number of full-term pregnancies, breastfeeding and menopausal status) since the number of cases are too few and because these factors are not real confounders and cannot affect the genotype. The POP variables (PFOS, PFOA, sum-PFSA, sum-PFCA, Sum-PCBs and sum-OCPs) were categorised as “low” or “high” based on the corresponding median values of control group. Additionally, these analyses were conducted with ln-transformed POP data as continuous variables. Women with the homozygous common genotypes and the lowest level of exposure to POPs were used as reference group in the analyses of the relation between SNPs, POPs and BC risk, respectively. For the interactions between genotypes and POP variables we also included the cross-product terms in the logistic regression models and assessed the Wald test statistic for these interaction terms.

All statistical analyses were performed using SPSS 19.0 statistical software (SPSS Inc., Chicago, IL, USA). A two-sided P value ≤ 0.05 was considered statistically significant in the analyses.

## Results

The demographic and baseline characteristic of the study population and the effects of POPs on BC risk have been previously reported [33] (see Additional file 1: Table S1). There were no significant differences among cases and controls in terms of median age, BMI and menopausal status. BC cases compared to their controls had lower number of full term pregnancies and serum cotinine level which reflects the current smoking status of the subjects.

### Allele and genotype frequencies in Greenlandic Inuit women and relationship with BC

Previously, we reported for the first time allele and genotype frequencies of CYP1A1 Ile 264Val, CYP1B1 Leu432Val and COMT Val158Met polymorphisms in Greenlandic Inuit [34]. The allelic frequencies of the CYP17 -34T>C (rs743572), CYP19 C > T (rs10046) and CYP19 (TTTA)n repeat have not been previously published for Inuit population.

Table 2 shows the genotype frequencies and allelic distribution of all studied SNPs in this study. For all SNPs, the genotype frequencies for both cases and controls were in HWE, with p values in the range 0.16 - 0.89 (not shown). In general for Greenlandic Inuit (control group), the frequency of CYP1A1 Val variant allele was 47.8%; CYP1B1 Val variant allele 14.9%, COMT low activity Met allele 59.7%; CYP17 A2 allele 53.5%; CYP19 C > T variant T allele 18.0%; CYP19 TTTA(11–13) 13.9% and the frequency of the BRCA1 founder mutation was 1.4%.

**Table 2:** Distribution of genotypes and the relationship between polymorphisms of studied genes and the risk of female breast cancer in Inuit

**Gene**	**Genotype**	**Case N (%)**	**Control N (%)**	**P (χ**^**2**^**)**	**Odds ratio (95% CI); p-value**		
					**Crude**	**Age adjusted**	**Age and cotinine adjusted**
*CYP1A1* (Ile462Val)	Ile/Ile	4 (13.3)	32 (28.3)	.241*	1.00 (Ref.)	1.00 (Ref.)	1.00 (Ref.)
	Ile/Val	17 (56.7)	54 (47.8)		2.52 (0.78-8.15); p = 0.123	2.52 (0.78-8.14); p = 0.124	**5.08 (1.19-21.6); p = 0.028**
	Val/val	9 (30.0)	27 (23.9)		2.67 (0.74-9.63); P = 0.134	2.66 (0.73-9.67); p = 0.138	3.31 (0.69-15.9); p = 0.135
	Ile/val + Val/val	26 (86.7)	81 (71.7)	.104**	2.57 (0.83-7.95); P = 0.102	2.56 (0.83-7.95); p = 0.103	**4.35 (1.08-17.5); p = 0.038**
	Val allele	35 (58.3)	108 (47.8)				
*CYP1B1* (Leu432Val)	Leu/Leu	26 (83.9)	79 (71.2)	.159	1.00 (Ref.)	1.00 (Ref.)	1.00 (Ref.)
	Leu/val	4 (12.9)	31 (27.9)		0.39 (0.13-1.21); p = 0.105	0.39 (0.13-1.22); p = 0.105	0.39 (0.094-1.45); p = 0.174
	Val/val	1 (3.2)	1 (0.9)		3.04 (0.18-50.3); p = 0.438	3.03 (0.18-50.1); p = 0.439	-
	Leu/val + Val/val	5 (16.1)	32 (28.8)	.174	0.48 (0.17-1.35); p = 0.161	0.47 (0.17-1.34); p = 0.160	0.39 (0.094-1.45); p = 0.174
	Val allele	6 (9.7)	33 (14.9)				
*COMT* (Val158Met)	Val/val	7 (22.6)	19 (16.8)	.504	1.00 (Ref.)	1.00 (Ref.)	1.00 (Ref.)
	Val/Met	11 (35.5)	53 (46.9)		0.56 (0.19-1.66); p = 0.299	0.56 (0.19-1.67); p = 0.300	0.65 (0.19-2.16); p = 0.476
	Met/met	13 (41.9)	41 (36.3)		0.86 (0.29-2.53); p = 0.783	0.87 (0.29-2.56); P = 0.871	0.65 (0.19-2.26); p = 0.498
	Val/Met-Met/Met	24 (77.4)	94 (83.2)	.441	0.69 (0.26-1.84); p = 0.461	0.69 (0.26-1.85); P = 0.463	0.65 (0.22-1.93); p = 0.435
	Met allele	37 (59.7)	135 (59.7)				
*CYP17* (−34 T > C)	TT (A1/A1)	12 (40)	24 (21.2)	.107	1.00 (Ref.)	1.00 (Ref.)	1.00 (Ref.)
	TC (A1/A2)	12 (40)	57 (50.4)		0.41 (0.16-1.05); p = 0.062	0.40 (0.16-1.03); p = 0.057	0.35 (0.12-1.05); p = 0.06
	CC (A2/A2)	6 (20)	32 (28.3)		0.36 (0.12-1.10); p = 0.073	0.35 (0.11-1.03); p = 0.067	**0.25 (0.063-0.95); P = 0.042**
	A1/A2 + A2/A2	18 (60)	89 (78.8)	.054	**0.39 (0.16-0.93); p = 0.033**	**0.38 (0.16-1.04); p = 0.030**	**0.34 (0.12-0.94); P = 0.038**
	A2 allele	24 (40)	121 (53.5)				
*CYP19* (C > T)	CC	23 (74.2)	79 (69.3)	.424	1.00 (Ref.)	1.00 (Ref.)	1.00 (Ref.)
	CT	8 (25.8)	29 (25.4)		0.95 (0.38-2.36); p = 0.908	0.94 (0.38-2.35) p = 0.944	1.29 (0.45-3.71); p = 0.639
	TT	0 (0)	6 (5.3)		-	-	-
	CT + TT	8 (25.8)	35 (30.7)	.662	0.79 (0.32-1.95); p = 0.608	0.78 (0.32-1.93); P = 0.594	1.13 (0.39-3.23); p = 0.821
	T allele	8 (12.9)	41 (18)				
*CYP19* (TTTA)n	F (7–10)	27 (87.1)	76 (75.2)	.317	1.00 (Ref.)	1.00 (Ref.)	1.00 (Ref.)
	SF	4 (12.9)	22 (21.8)		0.51 (0.16-1.62); p = 0.255	0.50 (0.16-1.58); p = 0.236	0.70 (0.2-2.45); p = 0.577
	S (11–13)	0 (0)	3 (3.0)		-	-	-
	SF + S	4 (12.9)	25 (24.8)	.217	0.45 (0.14-1.41); p = 0.17	0.44 (0.14-1.39); p = 0.160	0.70 (0.20-2.45); p = 0.577
	S allele	4 (6.5)	28 (13.9)				
*BRCA1* (Cys39Gly)	Cys/Cys	28 (90.3)	106 (97.2)	.122	1.00 (Ref.)	1.00 (Ref.)	1.00 (Ref.)
	Cys/Gly	3 (9.7)	3 (2.8)		3.79 (0.73-19.7); p = 0.115	4.29 (0.77-23.9); p = 0.097	5.14(0.45-58.9); p = 0.189
	Gly /Gly	0 (0)	0 (0)				
	Gly allele	3 (4.8)	3 (1.4)				

*p-value for comparison between the 3 genotypes (wt/wt; wt/var; var/var) using chi-2 analysis.

**p-value for comparison between the wt/wt and wt/var+var/var (dominant model).

Boldface indicate significance at p<0.05.

We compared the distribution of the SNPs between cases and controls and the association to BC (in models adjusted for age and cotinine) (Table 2). The χ^2^ tests for distribution showed no significant differences between cases and controls in most of the genotype frequencies except for borderline significant differences in CYP17 -34T>C (A1> A2) between cases and controls. The presence of at least one CYP17 A2 variant allele (A1/A2 + A2/A2) was borderline significant lower in cases (60% in cases vs. 79% in controls; p = 0.054). Although not significantly, the BC cases tend to have higher frequency of the CYP1A1 variant Val allele (57% in cases vs. 48% in controls), lower frequency of the CYP1B1 variant Val allele (9.7% in cases vs. 15% in controls), lower frequency of the CYP19 (C> T) variant T allele (13% vs. 18%) and lower frequency of the CYP19 (TTTA) _≥10_ allele (6.5% vs. 14%). We observed a strong correlation between long alleles of the CYP19 (TTTA)n polymorphism and the CYP19 T allele of rs10046 SNP: The longer (TTTA)n allele was significantly more common in individuals with the T allele of rs10046 SNP (r_s_ = 0.79; p < 0.0001).

In unconditional logistic regression analysis, we found that women with at least one CYP1A1 variant Val allele (Ile/Val + Val/Val) had a significantly increased risk of BC (adjusting for age + cotinine) compared to women with the common homozygous Ile/Ile genotype as reference (adjusted OR: 4.35; 95% CI: 1.08-17.4; p = 0.038) (Table 2).

Compared to women with the common homozygous A1/A1 genotype a significantly lower risk of BC was observed among women with at least one variant CYP17 A2 allele (adjusted OR: 0.34; 95% CI: 0.12-0.94; p = 0.038) (Table 2). Upon adjustment for age and cotinine the OR was still nearly the same and significant.

Compared to controls, the BRCA1 founder mutation allele was overrepresented among BC cases (cases: 4.8% vs. controls: 1.4%) and women with the BRCA1 founder mutation had a non-significant (because of low numbers) elevated risk of developing BC compared to those without this mutation (adjusted OR: 5.14; 95% CI: 0.45-58.87; p = 0.189). Adjusting for age and cotinine increased the OR to 5.14 (Table 2).

Further analyses by stratification by menopause status showed among premenopausal women a significant association between CYP1A1 variant Val allele and BC risk (OR: 5.180, 95% CI: 1.0-26.5) (Table 3). In addition, among premenopausal women a significant inverse association between the CYP1B1 variant Val allele and BC risk was observed (OR: 0.115, 95% CI: 0.01-1.00; p = 0.05). For the other genes no differences in ORs were seen between the strata of menopause status.

**Table 3:** Logistic regression stratified by menopause status for relationship between polymorphisms and the risk of female breast cancer in Inuit

**Variables**	**(Ca/Co) N**	**Premenopause**			***p***	**n**	**Postmenopause**			***p***
		OR^*^	**95% CI**			**(Ca/Co) N**	OR^*^	**95% CI**		
*CYP1A1* (Ile/Ile vs Ile/Val + Val/Val)	2/17 vs 14/25	5.18	1.01	26.5	0.048	2/15 vs 12/56	1.47	0.28	7.65	0.65
*CYP1B1* (leu/Leu vs Leu/Val + Val/Val)	16/28 vs 1/13	0.12	0.013	1.00	0.050	10/52 vs 4/19	1.56	0.40	6.09	0.52
*COMT* (Val/Val vs Val/Met + Met/Met)	3/8 vs 14/34	1.19	0.27	5.28	0.82	4/11 vs 10/60	0.53	0.13	2.20	0.38
*CYP17* (A1/A1 vs A1/A2 + A2/A2)	7/11 vs 9/31	0.42	0.12	1.43	0.16	5/12 vs 9/57	0.47	0.12	1.85	0.28
*CYP19 (C > T)* (CC vs CT + TT)	13/31 vs 4/11	0.79	0.21	3.04	0.74	10/48 vs 4/24	0.94	0.24	3.60	0.93
*CYP19 (TTTA)n* (F/F vs F/S + S/S)^#^	14/33 vs 3/7	0.95	0.21	4.27	0.94	13/43 vs 1/18	0.21	0.025	1.79	0.15

Ca: case, Co: control.

^*^Adjusted for age but not cotinine because of small sample size.

^#^F: 7–10 TTTA repeats; S: 11–13 TTTA repeats.

### Combined effect of polymorphism and serum POPs

Recently, we reported significant positive associations between serum levels of total PCBs and PFASs and BC in Greenlandic Inuit women for the same study group [33]. The BC cases had significantly higher concentrations of PFASs and also PCBs in the highest quartile, indicating that the level of serum PFASs and PCBs might be risk factors in the development of BC in Inuit.

The association between serum levels of PFASs and legacy POP variables (PCBs and OCPs) and risk of BC in Inuit women was evaluated after stratification by genotypes for the assessment of gene–environment interaction (Table 4) as well as using the POP data as continuous variables (see Additional file 1: Table S2).

**Table 4:** Odds ratios of breast cancer and 95% confidence intervals associated with PFOS and PFOA among breast cancer patients and controls

**Variables**		**N (Ca/Co)**	**Crude**		***p***	**N (Ca/Co)**	**Age adjusted**		***p***	**N (Ca/Co)**	**Age + cotinine adjusted**		***p***
			**OR**	**95% CI**			**OR**	**95% CI**			**OR**	**95% CI**	
***CYP1A1***	**PFOS**												
Ile/Ile	Low	1/17	1 (Ref)			1/17	1 (Ref)			0/17	1 (Ref)		
Ile/Ile	high	3/11	4.64	0.43-50.4	.208	3/11	6.33	0.35-114.1	.211	3/11			
Ile/Val + Val/Val	Low	2/32	1 (Ref)			2/32	1 (Ref)			2/32	1 (Ref)		
Ile/Val + Val/Val	High	24/36	10.67	2.34-48.7	.002	24/36	12.4	2.57-59.9	.002	20/36	12.1	1.29-115	.029
***CYP1B1***													
Leu/Leu	Low	3/28	1 (Ref)			3/28	1 (Ref)			2/28	1 (Ref)		
Leu/Leu	High	23/38	5.6	1.5-20.7	.009	23/38	7.3	1.80-29.4	.005	20/38	11.2	1.8-71.1	.011
Leu/val + Val/Val	Low	0/20	1 (Ref)			0/20	1 (Ref)			0/20	1 (Ref)		
Leu/val + Val/Val	High	5/8	0 (12.5)			5/8				4/8			
***COMT***													
Val/Val	Low	1/9	1 (Ref)			1/9	1 (Ref)			0/9	1 (Ref)		
Val/Val	High	6/8	6.75	0.66-68.8	.107	6/8	7.13	0.65-77.6	.107	5/8			
Val/Met + Met/Met	Low	2/40	1 (Ref)			2/40	1 (Ref)			2/40	1 (Ref)		
Val/Met + Met/Met	High	22/39	11.28	2.48-51.2	.002	22/39	15.36	3.02-78.2	.001	19/39	16.8	1.68-167	.016
***CYP17***													
A1/A1	Low	0/12				0/12				0/12			
A1/A1	High	10/12				10/12				10/9			
A1/A2 + A2/A2	Low	3/37	1 (Ref)			3/37	1 (Ref)			2/37	1 (Ref)		
A1/A2 + A2/A2	High	15/37	5.00	1.34-18.7	.017	15/37	5.67	1.38-23.4	.016	13/36	18.2	1.67-198.8	.017
***CYP19_CT***													
CC	Low	3/34	1 (Ref)			3/34	1 (Ref)			1/33	1 (Ref)		
CC	High	20/29	7.86	2.11-29.2	.002	20/29	8.85	2.15-36.4	.003	14/27	9.6	1.48-62.4	.018
CT + TT	Low	0/15				0/15				0/15			
CT + TT	High	8/19				8/19				8/19			
***CYP19_TTTA***													
(TTTA)_8–10_	Low	3/35	1 (Ref)			3/35	1 (Ref)			2/35	1 (Ref)		
(TTTA)_8–10_	High	24/29	9.66	2.64-35.3	.001	24/29	8.92	2.33-34.2	.001	20/29	29.3	2.89-298	.004
(TTTA)_11–13_	Low	0/11				0/11				0/11			
(TTTA)_11–13_	High	4/13				4/13				4/12			
Ile/Ile	Low	1/15	1 (Ref)			1/15	1 (Ref)			1/15	1 (Ref)		
Ile/Ile	High	3/13	3.46	0.32-37.5	.307	3/13	4.26	0.24-75.3	.322	3/13	10.5	0.12-907	.303
Ile/Val + Val/Val	Low	6/33	1 (Ref)			6/33	1 (Ref)			5/33	1 (Ref)		
Ile/Val + Val/Val	High	20/35	3.14	1.12-8.79	.029	20/35	3.30	1.13-9.7	.030	17/35	3.58	0.81-15.8	.092
***CYP1B1***													
Leu/Leu	Low	8/27	1 (Ref)			8/27	1 (Ref)			6/27	1 (Ref)		
Leu/Leu	High	18/39	1.6	0.59-4.10	.369	18/39	1.6	0.56-4.70	.351	16/39	2.1	0.59-7.6	.25
Leu/val + Val/Val	Low	0/20	1 (Ref)							0/20			
Leu/val + Val/Val	High	5/8	0 (12.5)							4/8			
***COMT***													
Val/Val	Low	3/8	1 (Ref)			3/8	1 (Ref)			2/8	1 (Ref)		
Val/Val	High	4/9	1.19	0.20-6.99	.851	4/9	1.15	0.19-6.87	.882	3/9	8.32	0.29-235	.215
Val/Met + Met/Met	Low	5/40	1 (Ref)			5/40	1 (Ref)			4/40	1 (Ref)		
Val/Met + Met/Met	High	19/39	3.89	1.32-11.5	.014	19/39	4.68	1.42-15.5	.011	17/39	3.73	0.86-16.3	.080
***CYP17***													
A1/A1	Low	3/10	1 (Ref)			3/10	1 (Ref)			2/10	1 (Ref)		
A1/A1	High	9/12	2.50	0.53-11.8	.247	9/12	2.35	0.45-12.2	.309	8/12	0.84	0.093-0.60	.877
A1/A2 + A2/A2	Low	4/38	1 (Ref)			4/38	1 (Ref)			3/38	1 (Ref)		
A1/A2 + A2/A2	High	14/36	3.69	1.11-12.3	.033	14/36	4.00	1.10-14.5	.035	12/36	8.79	1.22-63.5	.031
***CYP19_CT***													
CC	Low	7/32	1 (Ref)			7/32	1 (Ref)			5/32	1 (Ref)		
CC	High	16/31	2.36	0.85-6.55	.099	16/31	2.21	0.705-6.95	.173	13/31	2.44	0.57-10.4	.227
CT + TT	Low	1/16	1 (Ref)			1/16	1 (Ref)			1/16	1 (Ref)		
CT + TT	High	7/18	6.22	0.69-56.2	.104	7/18	7.18	.737-67.0	.090	7/18	4.99	0.34-74.7	.244
***CYP19_TTTA***													
(TTTA)_8–10_	Low	8/33	1 (Ref)			8/33	1 (Ref)			6/33	1 (Ref)		
(TTTA)_8–10_	High	19/31	2.53	0.97-6.61	.058	19/31	2.07	.753-5.71	.158	16/31	3.28	0.74-14.6	.119
(TTTA)_11–13_	Low	0/12	1 (Ref)			0/12	1 (Ref)			0/12	1 (Ref)		
(TTTA)_11–13_	High	4/12	0			4/12				4/12			

Ca: case, co: control.

In general, high serum PFAS levels (above the median distribution in the control group), increased the BC risk in all the genotype strata considered, indicating that PFAS exposure was a consistent risk factor for BC.

The risk of BC was not significantly associated with exposure to PCBs and OCPs, regardless of genotype for all investigated SNPs. This risk of BC associated to high serum PFOS levels was increased by approximately 2 fold in women with at least one variant CYP1A1 Val allele compared to women homozygous for the common Ile allele. The same association was found for COMT, where the presence of at least one variant COMT Met allele, and high PFOS and PFOA serum levels significantly increased the ORs of BC risk. Furthermore, women with the common CYP17 A1 allele and high levels of PFOS had higher risk for BC compared to women homozygous for the variant A2 allele. The risk of BC associated with PFOA was higher and significant among women with at least one CYP17 A2 allele (A1A2 + A2A2) compared to women homozygous for the A1 variant.

After stratification by CYP1B1 genotype, no cases were observed among those with low PFAS levels and carrier of at least one variant CYP1B1 Val allele, and therefore the ORs could not be determined. However, when the group (including case and controls) with the common CYP1B1 Leu/Leu genotype and low PFAS level was used as reference group instead, the ORs were the same for individuals with high PFAS exposure regardless of their CYP1B1 genotype (Leu/Leu versus Leu/Val + Val/Val) (not shown). Similarly, the genotype status of the CYP19 C > T SNP did not change the risk of BC associated to high PFASs (Table 4).

Despite the different risk of BC associated to PFOS/PFOA in different strata of gene polymorphisms we did not found a significant interaction between the PFASs variables and gene polymorphisms, when introducing the interaction terms into the model.

## Discussion

In the present case–control study of BC, six SNPs in key genes involved in xenobiotic metabolism as well as in oestrogen synthesis and metabolism and BRCA1 founder mutation were genotyped to explore the possible relationship of these polymorphisms in the development of BC in Greenlandic Inuit women.

Additionally, we evaluated the potential combined effects of these SNPs and environmental contaminant exposure. Our results showed higher tendency of BRCA1 founder mutation in cases than in controls and significant associations between BC and SNPs in CYP1A1 and CYP17. Generally, high serum PFAS levels was a consistent risk factor of BC, and in combination with having at least one variant CYP1A1 Val allele, or one variant COMT Met allele, or the common CYP17 A1 allele, the PFAS-associated BC risk increased, indicating that these polymorphisms can act as modulators of the development of BC related to environmental exposure.

Mutations in BRCA1 and BRCA2 are responsible for a large proportion of hereditary BC which account for approximately 8-10% of BC cases [35]. In the present study, BC cases had a higher frequency of the Greenlandic BRCA1 founder mutation compared with their controls (4.8% in cases vs. 1.4% in controls; Table 2); however, the BRCA1 mutation alone could not explain the frequency of BC cases in our study population. In the general Greenlandic population, the overall carrier frequency of BRCA1 founder mutation is 1.6%, but the frequency differs geographically from 0.6% at the West coast to 9.7% in the previously isolated populations at the East coast [36].

High and prolonged exposure to oestrogens plays a central role in breast carcinogenesis, by stimulating cell growth through oestrogen receptor pathway and by formation of genotoxic and mutagenic metabolites [37]. Therefore, functional SNPs which can change the levels of oestrogen or its metabolite have been extensively investigated in different populations, and controversial data are reported. Several factors might explain the controversy, such as interaction with other know BC risk factors, difference in polymorphism modifying the susceptibility of different ethnic groups to e.g. exposure to environmental contaminants. Inuit are genetically different from Caucasians in several genes [34,38] being more similar to East Asians, like Chinese. Moreover, Arctic Inuit e.g. Greenlandic have some of the highest exposure and thus body burden of environmental contaminants such as POPs. To our knowledge no studies have been reported for Inuit on gene polymorphisms, gene-environment interaction for genes involved in xenobiotic and oestrogen metabolism and associations with cancer risk.

In the present study, we report for the very first time the allelic frequencies of CYP17 -34T>C, CYP19 T > C and CYP19 (TTTA)n repeat in Greenlandic Inuit women, and previously we did report the allelic distribution of CYP1A1 Ile 264Val, CYP1B1 Leu432Val and COMT Val158Met polymorphisms in Greenlandic Inuit [34]. The frequency of the CYP17 -34T>C A2 allele (54%) in the present study was similar to those reported for Chinese (56%) [39], but higher than those reported for Japanese (43%) and Caucasians (37% in UK) [40]. The frequency of the CYP19 C > T variant T allele in the Inuit control group (18%) was relatively low and different from those reported for other populations such as Chinese and Caucasians (46%); but more similar to African-American (26.2%) [41]. For CYP19 (TTTA)n repeat we observed higher proportion of shorter repeats relative to higher repeats in line with what has been reported for Asians [28].

The CYP1A1 Ile462Val polymorphism was independently associated with increased risk of BC and significantly upon adjustment for current smoking (cotinine). It has been suggested that individuals with the CYP1A1 variant Val allele having increased inducibility and AHH enzymatic activity may produce more carcinogenic metabolites and thus more susceptible to developing BC. In Caucasians the variant CYP1A1 Val/Val genotype was suggested to be associated with a higher risk of BC [17], whereas in Chinese and Japanese this amino acid substitution was associated with other types of cancer, as lung cancer [42]. Several studies have shown a significant association between the CYP1A1 polymorphism and BC risk only in smokers [43,44], which could be related to the reported increase in inducibility of CYP1A1 polymorphism in smokers [9]. However, the association to BC was not seen in all women, suggesting that the variant CYP1A1 allele affects the BC risk by modifying the metabolism of the environmental carcinogens rather than oestrogens. In addition, based on in vitro data suggesting that variant forms of CYP1A1 are more inducible by PCBs, it has been hypothesised that there could be a combined effect between PCB levels and the variant CYP1A1 Val polymorphisms and increased BC risk. Previously it was reported a significantly increased postmenopausal BC risk, among women with high serum PCB levels (above the median for the control group) and with at least one Val allele [31]. Other studies have reported that the BC risk was significantly elevated among women with high PCB levels and the variant CYP1A1 polymorphisms who had ever smoked but not for those who had never smoked [30,31]. Another mode of action for PCBs was suggested e.g. the metabolism of PCBs by CYP1A1 to produce free radical and subsequently oxidative DNA damage in breast tissue [45].

However, our data did not support this observation for Greenlandic Inuit women exposed to high serum PCB levels, but for exposure to PFASs including PFOS. In our study population, the presence of the CYP1A1 Val allele increased the risk of BC associated to PFASs/PFOS. It is unknown whether PFOS, like some PCBs, is able to induce CYP1A1 gene via AhR that might lead to activation of procarcinogenic compounds and also oxidative metabolism of oestrogens [32]. PFOS and PFOA were reported to increase the CYP1A1 expression in salmon kidney and liver [46], and PFOS significantly further increased the TCDD-induced AhR activity in rat Hepatoma cells [47]. Recently, we also investigated the effect of seven PFASs on AhR, and found that only PFDoA and PFDA did transactivate AhR in mouse Hepatoma cells [48]. The effect of PFASs in human cells is unknown and warrants further investigation. Therefore, it seems biological plausible that there can be an interaction between CYP1A1 and PFASs on the risk of BC. Future studies are needed to confirm our data for Inuit women.

Many studies have investigated the effects of CYP1B1 polymorphisms in modifying BC risk [16], due to its role in production of 4-OH-E2, a potential carcinogenic estrogen metabolite, and the activation of a number of environmental carcinogens. Associations between the CYP1B1 Leu432Val polymorphism and BC risk seems to be race-specific as the homozygous variant CYP1B1 Val/Val genotype has been associated with BC in Caucasian women [49], while the wild type Leu/Leu genotype was associated with BC in Chinese postmenopausal women [50]. In Inuit population the Val/Val genotype was rare (under 1% for the control group). Although not significantly, Inuit BC cases had higher frequency of Leu/Leu compared to their controls, which is consistent with the results observed for Chinese women [50]. The amino acid change from Leu to Val increases the 4-OH enzyme activity of the CYP1B1 [51], whereas the AHH activity to form reactive metabolites from procarcinogens (e.g. PAHs and heterocyclic aryl amines) was shown to be slightly higher in the Leu432 form [14]. Therefore, it is plausible that Leu/Leu carriers exposed to high concentrations of POPs are consequently exposed to more reactive metabolites and thus more at risk for BC.

The cellular balance between phase I and phase II enzymes can be critical for the susceptibility to exposure to E2 cathechol metabolites. Many epidemiological studies have investigated the association of the phase II COMT variant (Met/Met) with BC, but the results are inconclusive [20,21]. Although most studies showed no association, some studies did find significant associations between the variant COMT Met allele and risk of BC [21]. In our study we did not observe any significant association to COMT Val158Met polymorphism. Human studies have suggested that high levels of CYP1B1 expression and low levels of COMT expression in adjacent non-tumour tissue were associated with an increased risk of BC [52] and other E2-responsive cancers [53,54]. However, it is also conceivable to speculate that a combination of a low-activity CYP1B1 (carrying the 432Leu allele) with a high activity COMT (carrying the common 158Val allele) may play a protective role for Inuit, since it might result in lower levels of catechol oestrogen and the subsequent oxidative damage in the cells. A study investigating the combined effect of high-risk genes for CYP1B1, COMT, glutathione S-transferase pi (GSTP1) and manganese superoxide dismutase (MnSOD) on BC risk in Caucasian postmenopausal women reported that the presence of both the CYP1B1 high activity Val and COMT low activity Met was associated with an increased risk of BC (OR 2.0, 95% CI 1.1–3.5) [55]. We also attempted to analyse the combined effect of the genes on BC risk, but did not get any significant results, which might be explained by the limited size of our study and the low frequency of CYP1B1 Val carriers, or different of ethnicity.

Among the Greenlandic Inuit women, the CYP17 variant A2 allele was associated with decreased risk of BC. A2 variant of CYP17 rs743572 SNP has been postulated to cause differences in circulating hormone levels and possibly influence the risk of BC [22,24,25]. However, these findings were not supported by other studies [27,56,57]. In postmenopausal Chinese women the CYP17 A1/A2 genotype was found to associate with increased risk of BC [39] compared with A1/A1 genotype. Similar to our result, in a Finnish population a tendency of lower risk of BC risk was found for premenopausal women carrying at least one CYP17 A2 allele [58]. CYP17 A2 was also reported to inversely associate with endometrial cancer risk among “Nurses’ Health Study” cohort [59]. A Meta analyses showed that, although not significantly, A2 was positively associated with risk of BC in post-menopausal women, but inversely related to BC in pre-menopausal women [60]. In contrast, two other Meta analyses found no association between A1 or A2 allele and BC risk [61]. In our study the risk of BC associated with CYP17 polymorphism was the same for pre- and postmenopausal women.

The CYP19 activity determines the local oestrogen level and it is biologically reasonable to hypothesis that variations in this gene can result in different expression or change in aromatase activity, which can affect oestrogen levels. In the current study, we found no significant BC risk associated to the two investigated polymorphisms in CYP19 gene ((TTTA)_n_ and rs10046). However, we observed a tendency for inverse association for longer repeats (TTTA)_11–13_ with BC risk, supported by a meta-analysis restricted to Asians (OR = 0.752, 95% CI = 0.603–0.939) [28]. This observation need to be further investigated in a larger study group among Inuit. The CYP19 T variant (CT + TT) in rs10046 polymorphism has been associated to BC risk in some studies including Asians [39]. However, in our study the occurrence of the T allele was lower among cases. Moreover, the frequency of the T allele in the Inuit control group (18%) was much lower than those reported for Asians and Causations. We cannot give or find any explanation for this finding.

Generally, high serum PFAS levels was a consistent risk factor of BC in this study population and higher levels of PCBs were also found in BC cases [2]. As discussed in our previous paper [2], several in vivo and in vitro studies have indicated that PFASs may have the potential to disrupt endocrine homeostasis. In vivo studies have reported decrease in thyroid hormone and increase in oestradiol levels after exposure to PFASs including PFOS and PFOA (reviewed in [62]). Our recent in vitro data showed that PFOS, PFOA and PFHxS significantly induced the ER transactivity in human MVLN breast carcinoma cells [63] and several PFASs including PFOS and PFOA antagonised the thyroid hormone-induced GH3 cell proliferation (T-screen assay) [48]. PFOS and PFOA were also found to induce the proliferation of human MCF-7 BOS breast cancer cells in the E-screen assay [64] and to induce the estrogen-responsive biomarker protein vitellogenin in juvenile rainbow trout [65]. Future studies are needed to understand the possible roles of PFASs as endocrine disrupters on sex hormone homoeostasis and function and their role in breast cancer patogenesis.

A limitation of our study is the small sample size and thus the statistical power for the detection of subtle changes might be relatively weak. Because of the low population size in Greenland (approximately 57.000) the number of BC cases during the sampling time was relatively low although the cases in this study comprise 80% of all the cases detected during this period. Therefore, our results should be interpreted with caution and there is a need for larger study group to confirm our results.

Most previous reports found that the effect of low-penetrance polymorphisms of individual gene on the risk of breast cancer was minor. This is not fully surprising since breast cancer is a multifactorial disease that involves interaction between multiple genetic and environmental events. However, larger population studies are needed to detect statistical evidence for gene–gene and gene–environment interactions.

## Conclusions

In conclusion, our results suggest that polymorphisms in CYP1A1 (Val) and CYP17 (A1) are associated with increased BC risk among Inuit women and this risk is increased in women having higher serum levels of PFASs (PFOS/PFOA). Serum PFAS levels were a consistent risk factor of BC, but inter-individual polymorphic differences might cause variations in sensitivity to the effects of these POPs as a potential cause of BC in Inuit.

### Consent

Written informed consent was obtained from the patient for the publication of this report and any accompanying images.

## Abbreviations

AHH: Aryl hydrocarbon hydrolase; AhR: Hydrocarbon receptor; BC: Breast Cancer; BMI: Body Mass Index; CI: Confidence intervals; COMT: Catechol-O-methyltransferase; CYP17: 17α-hydroxylase and 17, 20-lyase; CYP19: Aromatase; CYP1A1: Cytochrome P450 1A1; E2: Oestradiol; HWE: Hardy-Weinberg equilibrium; Ile: Isoleucine; OCPs: Organochlorine pesticides; OR: Odds ratio; PAH: Polycyclic aromatic hydrocarbon; PCB: Polychlorinated biphenyl; PFAS: Perfluoroalkylated substance; PFCA: Perfluorocarboxylated acid; PFOA: Perfluorooctanoic acid; PFOS: Perfluorooctane sulfonate; PFSA: Perfluorosulfonated acid; PHAH: Polyhalogenated aromatic hydrocarbon; POP: Persistent organic pollutant; SNP: Single nucleotide polymorphism; Val: Valine.

## Competing interests

The authors declare that they have no competing interest.

## Authors’ contributions

MG carried out the genotyping analyses, performed the statistical analyses and drafted the manuscript. ML participated in the study design, established the database and reviewed the manuscript drafts critically. HE carried out the genotyping of BRCA1 Greenlandic founder mutation and CYP19 (TTTA)n repeats and reviewed the final manuscript draft. ECB-J conceived the study and made the overall study design, guided the analyses and reviewed the manuscript drafts critically. All authors read and approved the final manuscript.

## Supplementary Material

Additional file 1: Table S1 Demographic, lifestyle and reproductive characteristics of breast cancer patients and controls. **Table S2.** Odds ratios of breast cancer and 95% confidence intervals associated with ln-transformed PFOS and PFOA among breast cancer patients and controls (ln-PFOS and ln-PFOA as continuous variables). Click here for file
