# Supplementary material for: Polymorphisms in Phase I and Phase II genes and breast cancer risk and relations to persistent organic pollutant exposure: a case–control study in Inuit women
Source: Environ Health. 2014 Mar 16;13:19. doi: 10.1186/1476-069X-13-19 (PMC4234380; doi:10.1186/1476-069X-13-19)
Supplement: Additional file 1: Table S1 — Demographic, lifestyle and reproductive characteristics of breast cancer patients and controls. Table S2. Odds ratios of breast cancer and 95% confidence intervals associated with ln-transformed PFOS and PFOA among breast cancer patients and controls (ln-PFOS and ln-PFOA as continuous variables). [file 1476-069X-13-19-S1.pdf]

Additional Table 1. Demographic, lifestyle and reproductive characteristics of breast cancer patients and controls

	Cases				Controls				p value
Parameters	N (n)	median	95% CI	Min-max	N (n)	median	95% CI	Min-max	
**Demographic and lifestyle factors**									
Age (years)	31 (31)	50	46.1; 56.7	29.0-80.0	115 (115)	54	49.1; 53.5	18-66	0.34
< 50	17 (54.8%)				37 (32.2%)				
51-55	3 (9.70%)				26 (22.6%)				
56-59	3 (9.70%)				17 (14.8%)				
>60	8 (25.8%)				35 (30.4%)				
BMI (kg/m^2)	31 (13)	26.9	24.0; 29.1	16.5-34.4	115 (115)	26.4	26.4;27.2	16.6-43.4	0.69
<25	4	23.5	16.0; 22.3	16.5-25.0	42	23.0	21.7;23.0	16.1-25.0	0.84
25-29	8	27.6	26.6; 28.9	25.8-29.6	42	27.6	26.9;27.8	25.1-19.9	0.46
> 30	1	34.4	-	-	31	33.2	33.1; 35.9	30.3-43.4	-
n-3/n-6	31 (29)	0.5	0.4; 0.6	0.2-1.7	115 (115)	0.5	0.5; 0.7	0.1-2.2	0.31
Serum cotinine (ng / ml)	31 (28)	11.6	33.3; 140.0	0-600.0	115 (96)	120	121.0; 188.0	0.0-799.0	0.052
Smoking status	31 (26)				115 (115)				0.77
Never	5 (19.2%)				21 (18.3%)				
Former	3(11.5%)				20 (17.4%)				
Current	18 (69.2%)				74 (64.3%)				
**Reproductive factors**									
Total number of full term pregnancies	31(16)	2.0	1.7; 2.8	1-4	115 (89)	3.0	3.2; 4.1	0.0-11.0	**<0.0001**
Ever breastfed	31(17)				115 (85)				
Yes (%)	15 (88.2%)				76 (89.4%)				0.89
Menopausal status#	31				115				
Premenopausal (%)	17 (54.8%)				42 (36.5%)				0.098
Postmenopausal (%)	14 (45.2%)				73 (63.5%)				
Serum E2 (nmol / l)	31(24)	0.09	0.09; 0.23	0.03-0.51	115(59)	0.07	0.05; 0.16	0.01-1.59	0.10
Premenopausal	11	0.21	0.13; 0.34	0.03-0.44	11	0.13	0.05; 0.55	0.12-1.59	0.52
Postmenopausal	13	0.06	0.02; 0.17	0.03-0.51	48	0.07	0.06; 0.08	0.01-0.17	0.73

N: total number of subjects, n: number of subjects having information for the corresponding parameters.

BMI: body mass index

#: Menopausal status was unknown for 36 of the samples (11 cases and 26 controls): among these, 31 women were between 18-42 years old and 5 women between 66-80 years old. In this study, women who had missing information on menopausal status were designated premenopausal if they were between 18-42 years old, and considered postmenopausal if they were between 66-80 years old.

Additional Table 2. Odds ratios of breast cancer and 95% confidence intervals associated with ln-transformed PFOS and PFOA among breast cancer patients and controls (ln-PFOS and ln-PFOA as continuous variables)

Gene	Genotype	Exposure	N (ca/co)	Age adjusted	95% CI	p
CYP1A1 (Ile462Val)	Ile/Ile	PFOS	4/28	1.71	0.47-6.32	0.418
	Ile/Val+Val/Val	PFOS	26/68	2.63	1.46-4.75	0.001
	Ile/Ile	PFOA	4/28	1.01	0.26-3.88	0.993
	Ile/Val+Val/Val	PFOA	26/68	1.79	0.97-3.32	0.064
	Ile/Ile	PCBs	4/32	.68	0.12-3.76	0.663
	Ile/Val+Val/Val	PCBs	25/81	1.05	0.50-2.19	0.898
	Ile/Ile	OCPs	4/32	0.78	0.17-3.50	0.747
	Ile/Val+Val/Val	OCPs	25/81	1.72	0.76-3.89	0.193
CYP1B1 (Leu432Val)	Leu/Leu	PFOS	26/66	1.95	1.09-3.48	0.025
	Leu/val+Val/Val	PFOS	5/28	8.67	1.33-56.6	0.024
	Leu/Leu	PFOA	26/66	1.15	0.64-2.07	0.645
	Leu/val+Val/Val	PFOA	5/28	10.10	1.25-81.3	0.030
	Leu/Leu	PCBs	26/79	0.97	0.47-2.02	0.937
	Leu/val+Val/Val	PCBs	4/32	0.39	0.04-3.59	0.408
	Leu/Leu	OCPs	26/79	1.50	0.67-3.36	0.320
	Leu/val+Val/Val	OCPs	4/32	1.08	0.20-5.70	0.931
COMT (Val158Met)	Val/Val	PFOS	7/17	2.70	0.83-8.79	0.100
	Val/Met+Met/Met	PFOS	24/79	2.65	1.44-4.89	0.002
	Val/Val	PFOA	7/17	1.10	0.34-3.40	0.909
	Val/Met+Met/Met	PFOA	24/79	1.88	0.98-3.60	0.055
	Val/Val	PCBs	6/19	1.96	0.33-11.6	0.457
	Val/Met+Met/Met	PCBs	24/94	1.00	0.49-2.05	0.994
	Val/Val	OCPs	6/19	2.64	0.32-21.1	0.368
	Val/Met+Met/Met	OCPs	24/94	1.53	0.72-3.25	0.267
CYP17 (−34T>C)	A1A1	PFOS	12/22	4.89	1.28-18.7	0.020
	A1A2+A2A2	PFOS	18/74	2.21	1.19-4.12	0.013
	A1A1	PFOA	12/22	2.11	0.73-6.01	0.166
	A1A2+A2A2	PFOA	18/74	1.48	0.76-2.89	0.252
	A1A1	PCBs	12/24	1.56	0.55-4.45	0.402
	A1A2+A2A2	PCBs	17/89	0.94	0.39-2.23	0.886
	A1A1	OCPs	12/24	1.74	0.53-5.67	0.361
	A1A2+A2A2	OCPs	17/89	1.68	0.68-4.15	0.259
CYP19 (C>T)	CC	PFOS	23/63	2.65	1.39-5.06	0.003
	CT+TT	PFOS	8/34	2.54	0.92-7.04	0.073
	CC	PFOA	23/63	1.37	0.71-2.63	0.346
	CT+TT	PFOA	8/34	2.47	0.82-7.42	0.106
	CC	PCBs	22/79	1.07	0.51-2.26	0.862
	CT+TT	PCBs	8/35	1.16	0.32-4.21	0.821
	CC	OCPs	22/79	1.55	0.70-3.41	0.279
	CT+TT	OCPs	8/35	1.90	0.45-7.99	0.380
CYP19_TTTA (TTTA)n	$(TTTA)_{8\text{-}10}$	PFOS	27/64	2.58	1.40-4.75	0.002
	$(TTTA)_{11\text{-}13}$	PFOS	4/24	41.9	0.42-4203	0.112
	$(TTTA)_{8\text{-}10}$	PFOA	27/64	1.30	0.72-2.34	0.381
	$(TTTA)_{11\text{-}13}$	PFOA*	4/24	1.88	0.48-7.33	0.363
	$(TTTA)_{8\text{-}10}$	PCBs	26/76	1.05	0.51-2.18	0.892
	$(TTTA)_{11\text{-}13}$	PCBs	4/25	1.54	0.23-10.3	0.657
	$(TTTA)_{8\text{-}10}$	OCPs	26/76	1.65	0.75-3.63	0.215
	$(TTTA)_{11\text{-}13}$	OCPs	4/25	1.97	0.27-14.3	0.504

Ca: case; Co: control ; CI: Confidence interval; OCPs: sum of 8 organochlorine pesticides; PCBs: sum of 12 PCBs
